# Supplementary material for: Structural basis of rapid actin dynamics in the evolutionarily divergent Leishmania parasite
Source: Nat Commun. 2022 Jun 15;13:3442. doi: 10.1038/s41467-022-31068-y (PMC9200798; doi:10.1038/s41467-022-31068-y)
Supplement: Supplementary file 1 — Supplementary Information [file 41467_2022_31068_MOESM1_ESM.pdf]

Supplementary Information

Kotila *et al.* 2022

Structural basis of rapid actin dynamics in the evolutionarily divergent *Leishmania* parasite

Supplementary Information includes:

-Supplementary Figs. 1-8

-Supplementary Table 1-3

Fig. S1

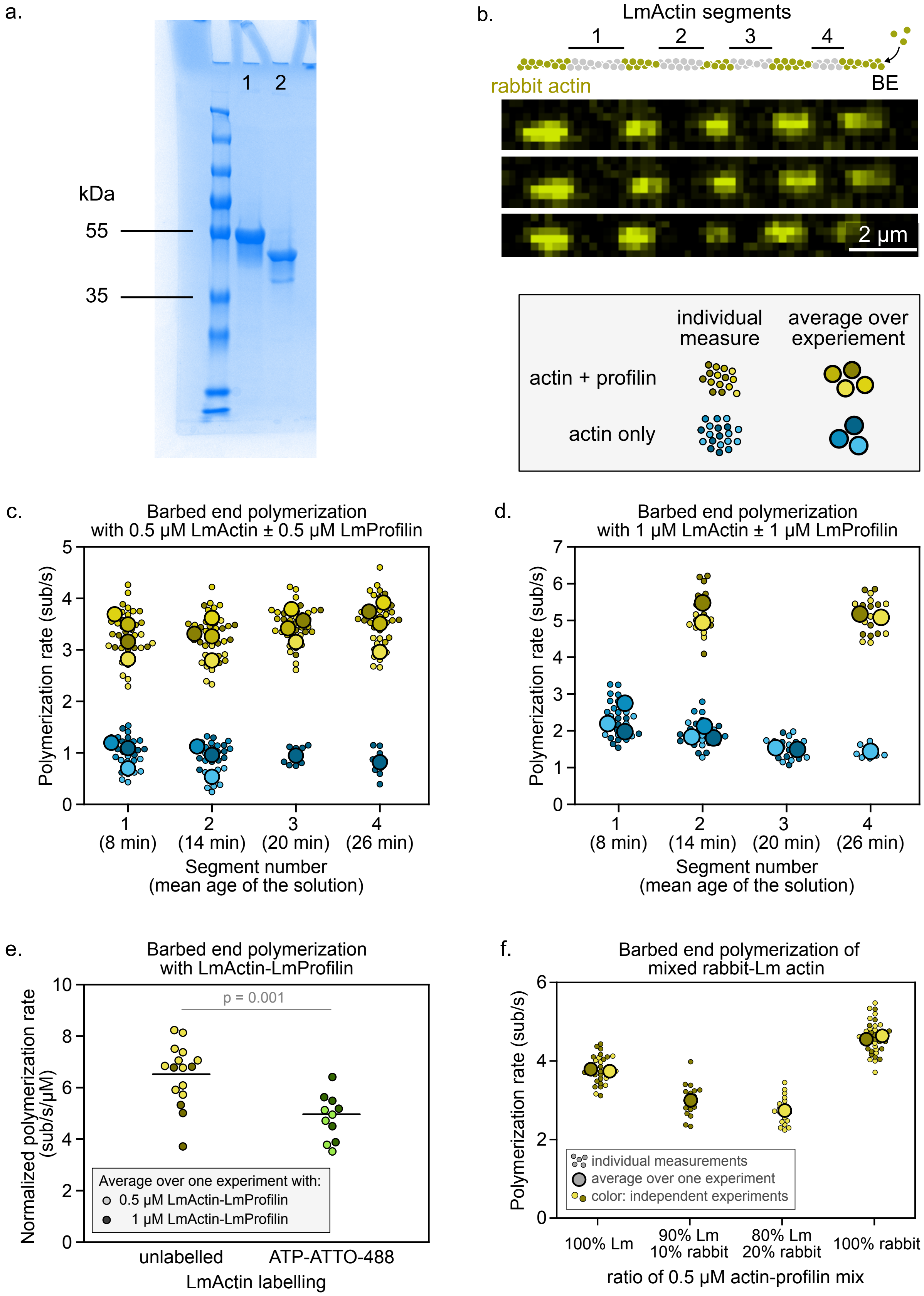

a.
1 2
kDa
55
35
b.
LmActin segments
1 2 3 4
rabbit actin
BE
2 μm
individual measure
average over experiment
actin + profilin
actin only
c.
Barbed end polymerization
with 0.5 μM LmActin ± 0.5 μM LmProfilin
Polymerization rate (sub/s)
1 (8 min) 2 (14 min) 3 (20 min) 4 (26 min)
Segment number
(mean age of the solution)
d.
Barbed end polymerization
with 1 μM LmActin ± 1 μM LmProfilin
Polymerization rate (sub/s)
1 (8 min) 2 (14 min) 3 (20 min) 4 (26 min)
Segment number
(mean age of the solution)
e.
Barbed end polymerization
with LmActin-LmProfilin
Normalized polymerization rate (sub/s/μM)
p = 0.001
Average over one experiment with:
0.5 μM LmActin-LmProfilin
1 μM LmActin-LmProfilin
unlabelled
ATP-ATTO-488
LmActin labelling
f.
Barbed end polymerization of
mixed rabbit-Lm actin
Polymerization rate (sub/s)
individual measurements
average over one experiment
color: independent experiments
100% Lm
90% Lm 10% rabbit
80% Lm 20% rabbit
100% rabbit
ratio of 0.5 μM actin-profilin mix

Supplementary Fig. 1. Purification, spontaneous nucleation and polymerization of LmActin. **(a)** Purity of *Leishmania* actin produced as β-thymosin fusion protein in Sf9 insect cells. Lane 1: β-thymosin-LmActin after Ni-NTA and gel filtration purification steps. Lane 2, LmActin after chymotrypsin cleavage of β-thymosin, and a single round of polymerization/depolymerization cycle. Please note a small amount of unspecifically cleaved product after depolymerization/polymerization steps. (**b**) Images of segmented filaments made of four LmActin segments polymerized from the same solution of 1 μM LmActin of increasing age. Green fluorescence: rabbit Alexa488-actin. BE: barbed end. (**c-d**) Evolution of the barbed end polymerization rate from an aging solution of unlabeled G-LmActin with (yellow) and without (blue) LmProfilin in F-buffer. The age of the solution corresponds to the time spent between mixing the G-actin stock into F-buffer, and polymerization of a given segment. For panel c: LmActin + LmProfilin: N=4, n=10. LmActin: N=3, n=10. For panel d: LmActin + LmProfilin: N=2, n=10. LmActin: N=3, n=10. **(e)** Impact of ATP-ATTO-488 labeling on the polymerization rate of 0.5 μM and 1 μM LmActin:LmProfilin (bright and dark data points, respectively). The polymerization rate was normalized by the actin-profilin concentration. Each data point corresponds to the average over 16-50 filaments (n) from an independent experiment (N). Horizontal line: average over all experiments. P-value was obtained from a two-sided t-test of independent samples (Python scipy.stats.ttest_ind function). For unlabeled LmActin: N=11, n=50, 40, 40, 50, 60, 20, 20, 20, 20, 20, 10 for 0.5 μM; N=5, n= 20, 20, 30, 40, 40 for 1 μM. For ATP-ATTO labeled LmActin: N=5, n=20, 20, 20, 20, 30 for 0.5 μM; N=6, n=30, 30, 20, 20, 16, 20 for 1 μM. **(f)** Barbed end polymerization rates from solutions containing 0.5 μM actin-profilin, and with various fractions of *Leishmania* and mammalian proteins. Small points correspond to individual measurements, and large ones are averages over one experiment. Data points with the same brightness were obtained from a single experiment, and with the same protein stocks. The indicated percentages correspond to the fractions of *Leishmania* and rabbit proteins in the solutions. For 100% LmActin N=2, n=20, 10; 90% LmActin, 10% rabbit N=1, n=20; 80% LmActin, 20% rabbit N=1, n=20; 100% rabbit actin: N=2, n=20, 20.

Fig. S2

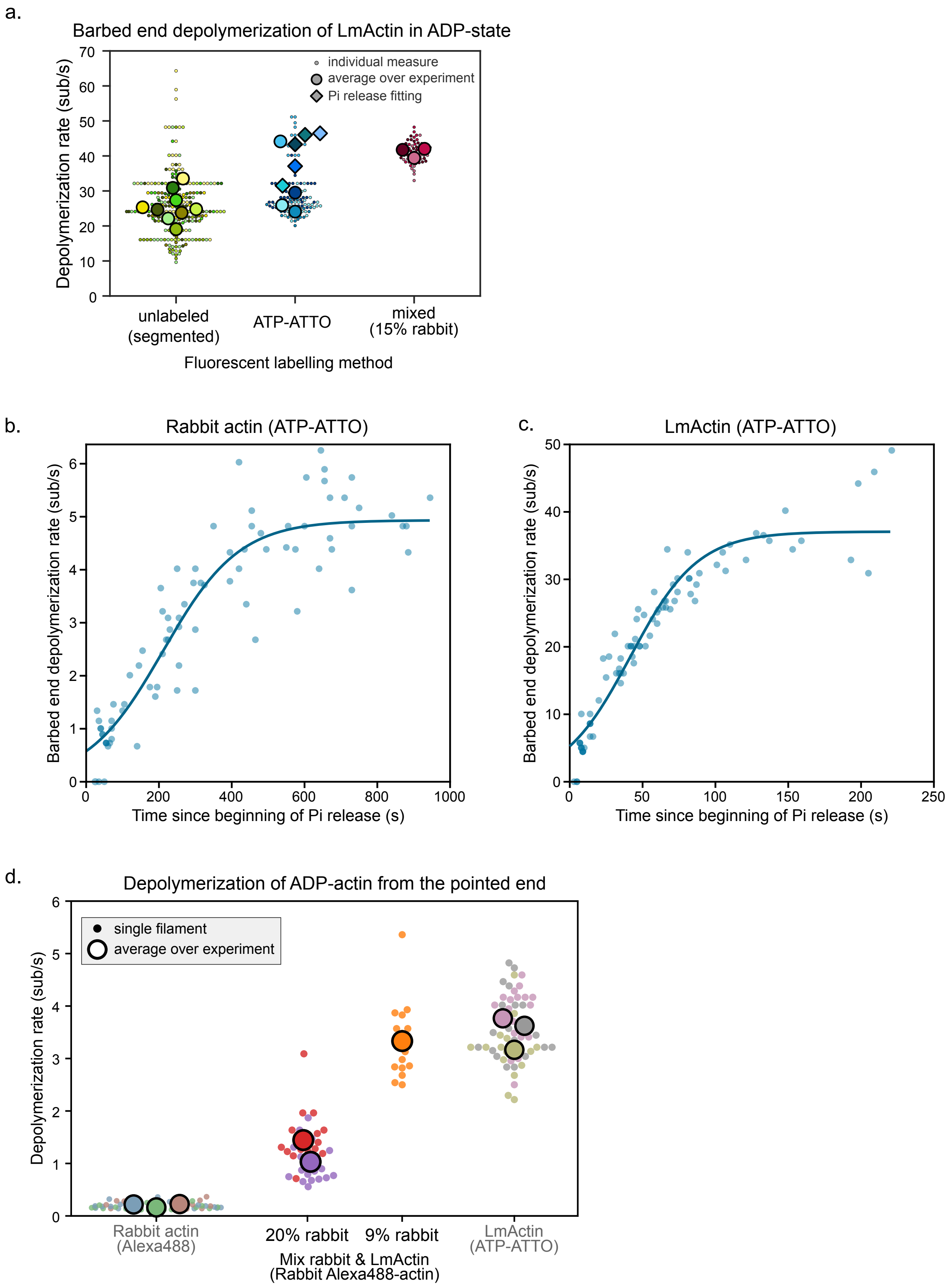
a.
Barbed end depolymerization of LmActin in ADP-state
individual measure
average over experiment
Pi release fitting
Depolymerization rate (sub/s)
unlabeled (segmented)
ATP-ATTO
mixed (15% rabbit)
Fluorescent labelling method
b.
Rabbit actin (ATP-ATTO)
Barbed end depolymerization rate (sub/s)
Time since beginning of Pi release (s)
c.
LmActin (ATP-ATTO)
Barbed end depolymerization rate (sub/s)
Time since beginning of Pi release (s)
d.
Depolymerization of ADP-actin from the pointed end
single filament
average over experiment
Depolymerization rate (sub/s)
Rabbit actin (Alexa488)
20% rabbit
9% rabbit
Mix rabbit & LmActin (Rabbit Alexa488-actin)
LmActin (ATP-ATTO)

Supplementary Fig. 2. Pi release and filament depolymerization of *Leishmania* and rabbit actin. (**a**) Impact of actin labelling method on the barbed end depolymerization rate of ADP-LmActin. Large circles represent averages over 20-40 filaments from one experiment. Diamond symbols represent ADP-actin depolymerization rates fitted from Pi release experiment (Fig. 2). "Mixed" filaments were polymerized from a single solution containing both Lm and rabbit actin. Based on the fluorescence intensity, we estimate that filaments were composed of 15% RbActin / 85% LmActin (see Methods). unlabeled: For unlabelled actin: N=9, n=30, 32, 32, 19, 40, 20, 20, 20, 20. For ATP-ATTO-labelled actin: N=9, n=20, 40, 20, 40, 71, 25, 73, 58, 85. For mixed filaments: N=3, n=20. (**b-c)** Barbed end depolymerization rate increases as filament ages. ADP-Pi actin filaments were first assembled in a buffer with excess of Pi and were then exposed from t=0 onward to standard F-buffer without excess phosphate buffer. Data points correspond to single measurements made manually on kymographs over one movie. The depolymerization rate increases as Pi is released and filaments become mostly ADP-actin. For curve fit see Methods (equation 1). For rabbit actin N=1, n=81; for LmActin N=1, n=85. (**d**) Pointed end depolymerization rate of actin filaments pre-polymerized from a solution of either pure LmActin, pure RbActin, or a mix of the two. In the latter case, the fraction of RbActin in filaments was estimated from the fluorescence signal (see Methods). Each color depict a single independent experiment. Small points are single measurements (n), and large ones represent the averages over the experiment. For RbActin N=3, n=20, 20, 10; 20% rabbit N=2, n=20; 9% rabbit N=1, n=17; LmActin N=3, n=20, 20, 16.

Fig. S3

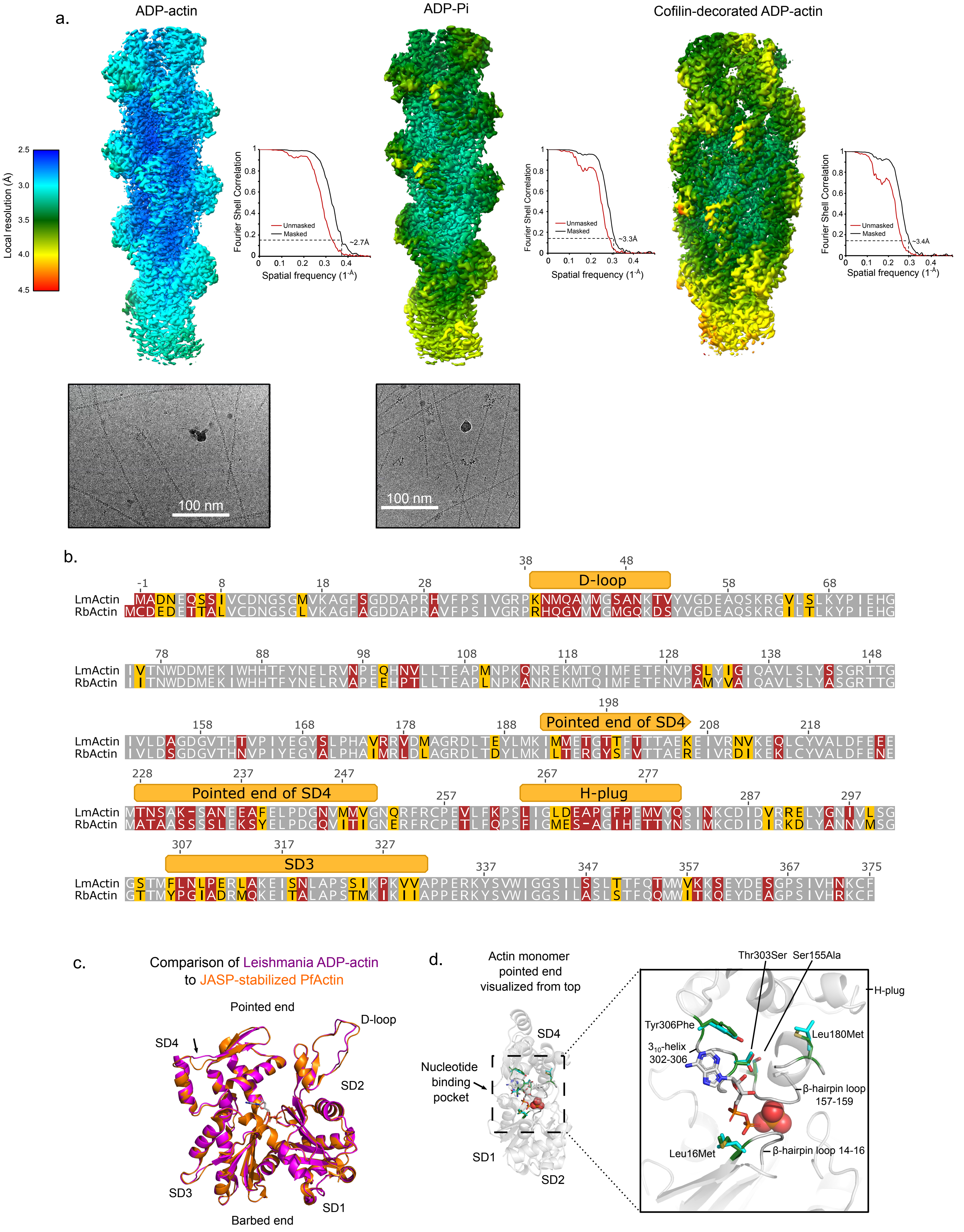

a.
ADP-actin
ADP-Pi
Cofilin-decorated ADP-actin
Local resolution (Å)
2.5
3.0
3.5
4.0
4.5
Fourier Shell Correlation
Spatial frequency (1/Å)
Unmasked
Masked
~2.7Å
~3.3Å
~3.4Å
100 nm
b.
D-loop
Pointed end of SD4
H-plug
SD3
LmActin MADNEQSSIVCDNGSGMVKAGFSGDDAPRHVFPSIVGRPKNMQAMMGSANKTVYVGDEAQSKRGVLSLKYPIEHG
RbActin MCDEDETTALVCDNGSGLVKAGFAGDDAPRAVFPSIVGRPRHQGVMVGMGQKDSYVGDEAQSKRGILTLKYPIEHG
LmActin IVTNWDDMEKIWHHTFYNELRVNPEQHNVLLTEAPMNPKQNREKMTQIMFETFNVPSLYIGIQAVLSLYSSGRTTG
RbActin IITNWDDMEKIWHHTFYNELRVAPEEHPTLLTEAPLNPKANREKMTQIMFETFNVPAMYVAIQAVLSLYASGRTTG
LmActin IVLDAGDGVTHTVPIYEGYSLPHAVRRVDMAGRDLTEYLMKIMMETGTTFTTTAEKEIVRNVKEQLCYVALDFEEE
RbActin IVLDSGDGVTHNVPIYEGYALPHAIMRLDLAGRDLTDYLMKILTERGYSFVTTAEREIVRDIKEKLCYVALDFENE
LmActin MTNSAK-SANEEAFELPDGNVMMVGNQRFRCPEVLFKPSLIGLDEAPGFPEMVYQSINKCDIDVRRELYGNIVLSG
RbActin MATAASSSSLEKSYELPDGQVITIGNERFRCPETLFQPSFIGMES-AGIHETTYNSIMKCDIDIRKDLYANNVMSG
LmActin GSTMFLNLPERLAKEISNLAPSSIKPKVVAPPERKYSVWIGGSILSSLTTFQTMWVKKSEYDESGPSIVHNKCF
RbActin GTTMYPGIADRMQKEITALAPSTMKIKIIAPPERKYSVWIGGSILASLSTFQQMWITKQEYDEAGPSIVHRKCF
c.
Comparison of Leishmania ADP-actin to JASP-stabilized PfActin
Pointed end
D-loop
SD4
SD2
SD3
SD1
Barbed end
d.
Actin monomer pointed end visualized from top
Nucleotide binding pocket
SD4
SD1
SD2
Thr303Ser
Ser155Ala
H-plug
Tyr306Phe
Leu180Met
3₁₀-helix 302-306
β-hairpin loop 157-159
Leu16Met
β-hairpin loop 14-16

Supplementary Fig. 3. CryoEM structures obtained in the study. (**a**) Local resolutions of the Relion post-process cryoEM maps automatically sharpened in Relion. Corresponding FSC plots are shown for each reconstruction. Examples of EM images for the ADP and ADP-Pi actins are shown below the cryoEM maps. Please note that the ADP-actin sample contains a mixture of bare and cofilin-decorated filaments (see 'Methods' for details of sample preparation and image processing). (**b**) Sequence alignment of rabbit muscle actin and *Leishmania major* actin. Annotated regions correspond to the structures in Fig. 4. (**c**) Structural comparison of the *Leishmania major* ADP- actin and jasplakinolide-stabilized *Plasmodium falciparum* actin. Actins superimpose nearly perfectly (RMSD 0.400, PDB=6TU4). (**d**) Comparison of the sequence conservation in the nucleotide binding pocket of actin between chicken muscle actin (cyan, PDB=6djn) and *Leishmania major* ADP-Pi state actin (green). Most of the substitutions are minor, but could alter the dynamics of the nucleotide binding pocket. Note that the nucleotide coordinating loops are interconnected to the H-plug region.

Fig. S4

a.

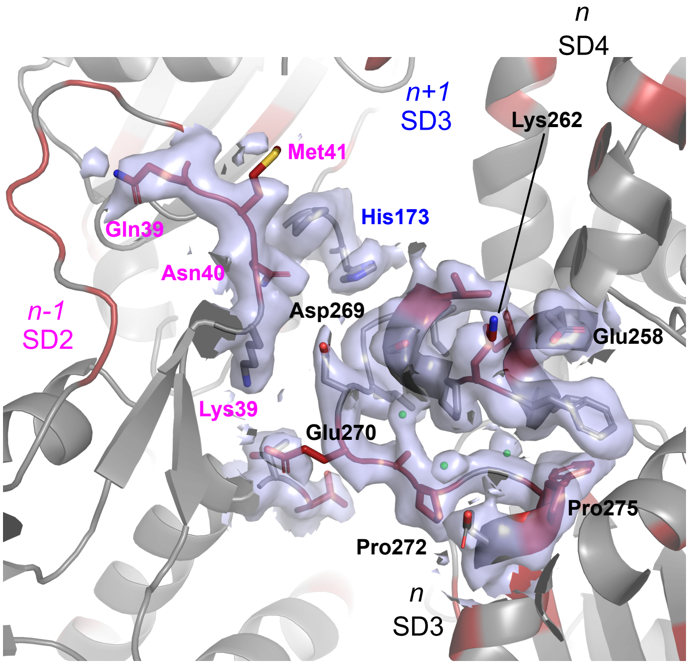

Lateral contacts in the center of the filament

b.

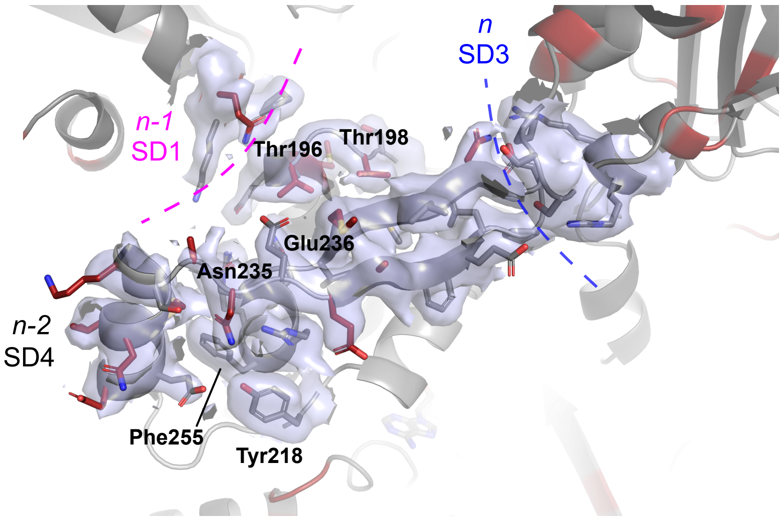

Longitudinal contacts between SD4, SD1 and SD3

c.

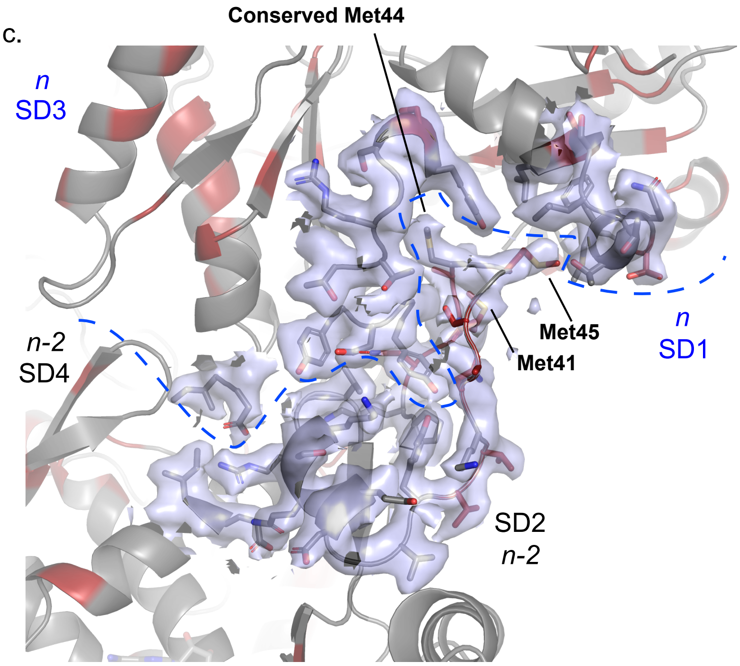

Longitudinal contacts between SD2 and barbed end

Supplementary Fig. 4. The intra- and intersubunit contacts of *Leishmania* actin filament overlaid with CryoEM experimental density. Contacting residues, which are of different amino acid class between the two actins (see: Fig. 3e) are shown in red. The corresponding cryoEM density is overlaid in light blue. Note that the amino acid labels are colored according to the subunit they belong to. The orientations of the presented views were selected for optimal clarity. (**a**) The lateral contacts between three subunits (n, n-1, n+1) from the center of the filament. (**b**) The pointed end longitudinal contacts formed between subdomain 4 of actin subunit n-2 and subunits n and n-1. (**c**) The pointed end longitudinal contacts formed between subdomain 2 of actin subunit n-2 and subunit n.

Fig. S5

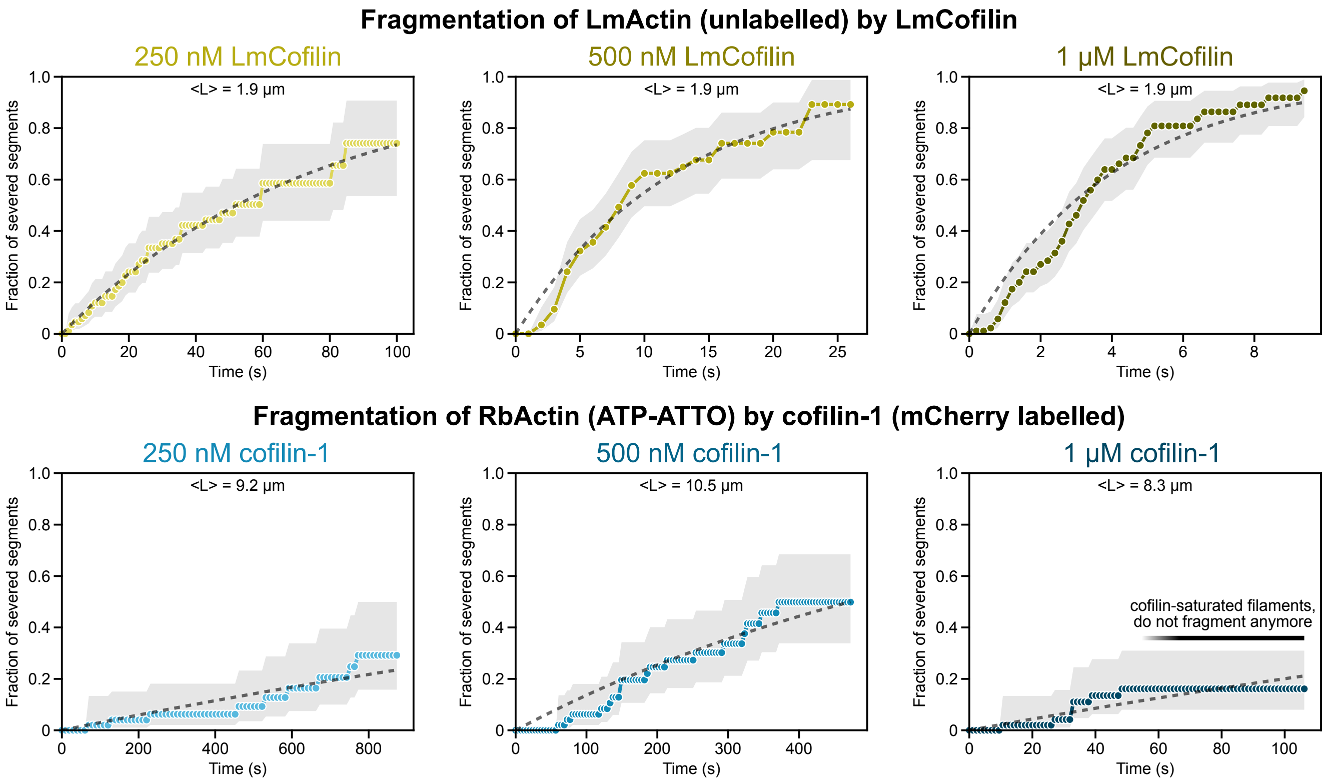
Fragmentation of LmActin (unlabelled) by LmCofilin
250 nM LmCofilin
500 nM LmCofilin
1 μM LmCofilin
<L> = 1.9 μm
Fraction of severed segments
Time (s)
Fragmentation of RbActin (ATP-ATTO) by cofilin-1 (mCherry labelled)
250 nM cofilin-1
500 nM cofilin-1
1 μM cofilin-1
<L> = 9.2 μm
<L> = 10.5 μm
<L> = 8.3 μm
cofilin-saturated filaments, do not fragment anymore

Supplementary Fig. 5. Fragmentation dynamics of actin filaments by ADF/cofilin compared between *Leishmania* and vertebrate proteins. LmActin (unlabelled, top) and RbActin (labelled with ATP-ATTO, bottom) were exposed from time t=0 onwards to 0.25-1 µM LmCofilin and mCherry-cofilin-1, respectively. For LmCofilin severing on LmActin, segmented filaments were made (Fig. 1c-e) and their severing was observed thanks to the departure of the downstream labeled RbActin segments. The fractions of un-severed actin segments were calculated as Kaplan-Meier survival fractions (Methods) and fitted with a single exponential (see Methods, equation 2) to estimate the global fragmentation rate (dotted line). <L> corresponds to the mean segment length, used to calculate the global fragmentation rate. Note the difference in time scales between experiments. Experiments with LmActin are well fitted by a single exponential suggesting that LmCofilin constantly binds F-actin and very quickly severs filaments. In contrast, the fragmentation of mammalian F-actin follows more complex dynamics. At low concentration (250 nM), cofilin-1 forms few domains that slowly elongate and severs filaments. At high concentration (1 µM and to a lesser extent 500 nM), cofilin-1 rapidly saturates filaments, preventing further fragmentation. In such a case, the global fragmentation rate is not well defined. Shaded gray surfaces: 95% confidence intervals. Dotted line: exponential fit. N=1, n=90 for 250 nM LmActin;, N=1, n=90 for 500 nM LmActin; N=1, n=90 for 1 µM LmActin; N=1, n=50 for 250 nM RbActin; N=1, n=50 for 500 nM RbActin; N=1, n=50 for 1 µM RbActin.

Fig. S6

a. Comparison between *Leishmania* cofilin and *S. cerevisiae* cofilin

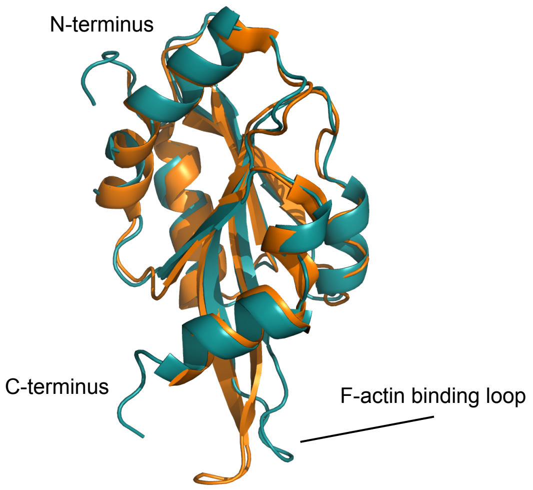

b. *Leishmania* actin binding surface against chicken cofilin

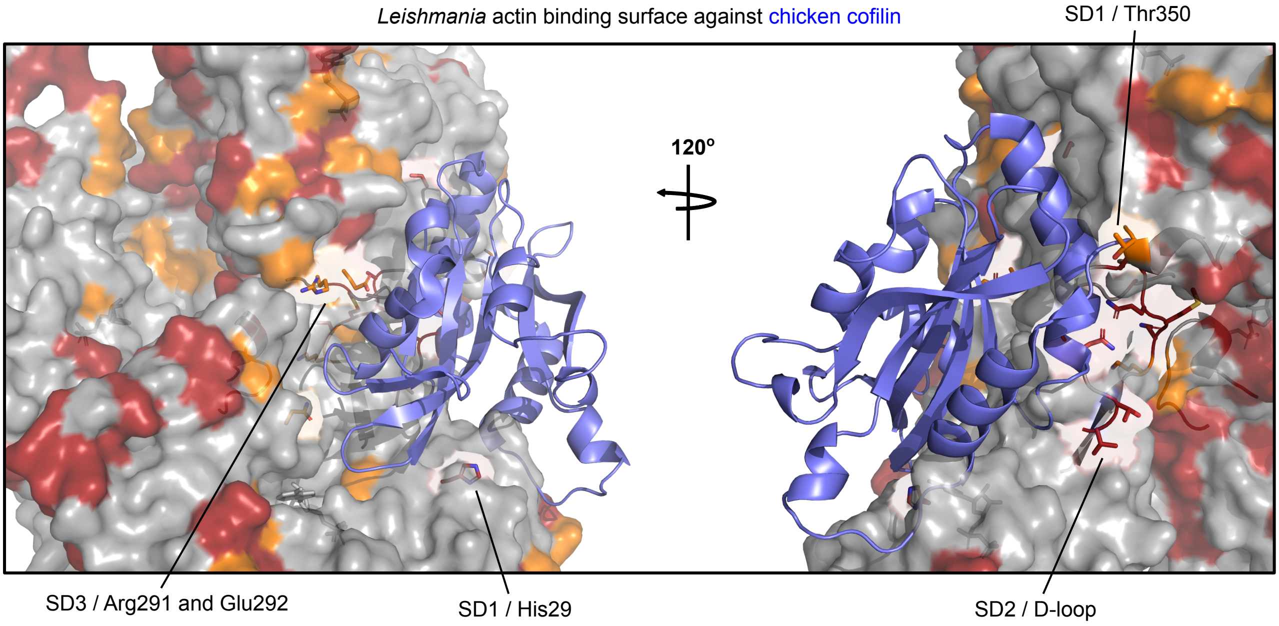

c. Severing of segmented filaments by LmCofilin. Severing only occurs at LmActin regions.

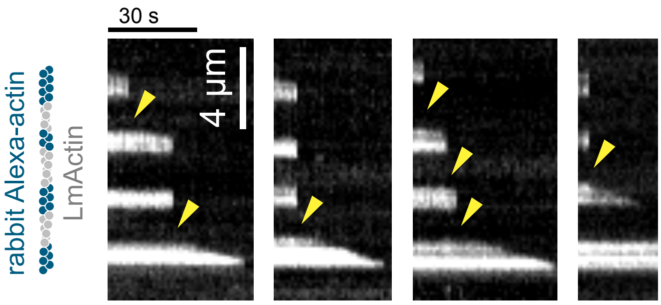

Supplementary Fig. 6. Cofilin conservation and binding surface on *Leishmania* actin. (**a**) Comparison between *Leishmania* cofilin and *S. cerevisiae* cofilin (PDB=1qpv) structures. Please note that the last four C-terminal residues were not built in yeast cofilin structure, but exist in the sequence thus making the C-terminus length identical to *Leishmania* cofilin. (**b**) Differences between Leishmania and rabbit actin sequences near the cofilin binding surface. Gray color indicates identical, orange similar, and red different amino acids in the corresponding positions between the two actins. The cofilin-binding sites are well-conserved, except for the D-loop region. (**c**) Fragmentation of segmented filaments by 500 nM LmCofilin from single experiment. Severing event most likely occur inside the LmActin segment. No severing events were observed within rabbit Alexa-actin segments.

Fig. S7

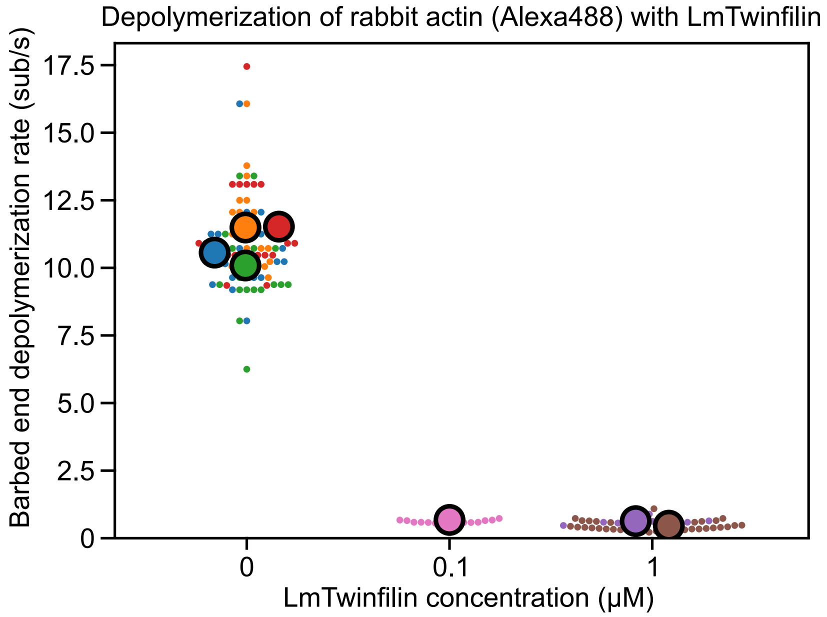

Depolymerization of rabbit actin (Alexa488) with LmTwinfilin
Barbed end depolymerization rate (sub/s)
17.5
15.0
12.5
10.0
7.5
5.0
2.5
0
0
0.1
1
LmTwinfilin concentration (μM)

Supplementary Fig. 7. Barbed end depolymerization rate of rabbit Alexa-ADP-actin in presence or absence of LmTwf. Large symbols represent averages over 15-30 individual measures (small symbols) from a single independent experiment. Symbol colours represent different experiments. N=4, n=20, 24, 24, 20 for 0 μM; N=1, n=20 for 0.1 μM; N=2, n=15, 40 for 1 μM.

Fig. S8

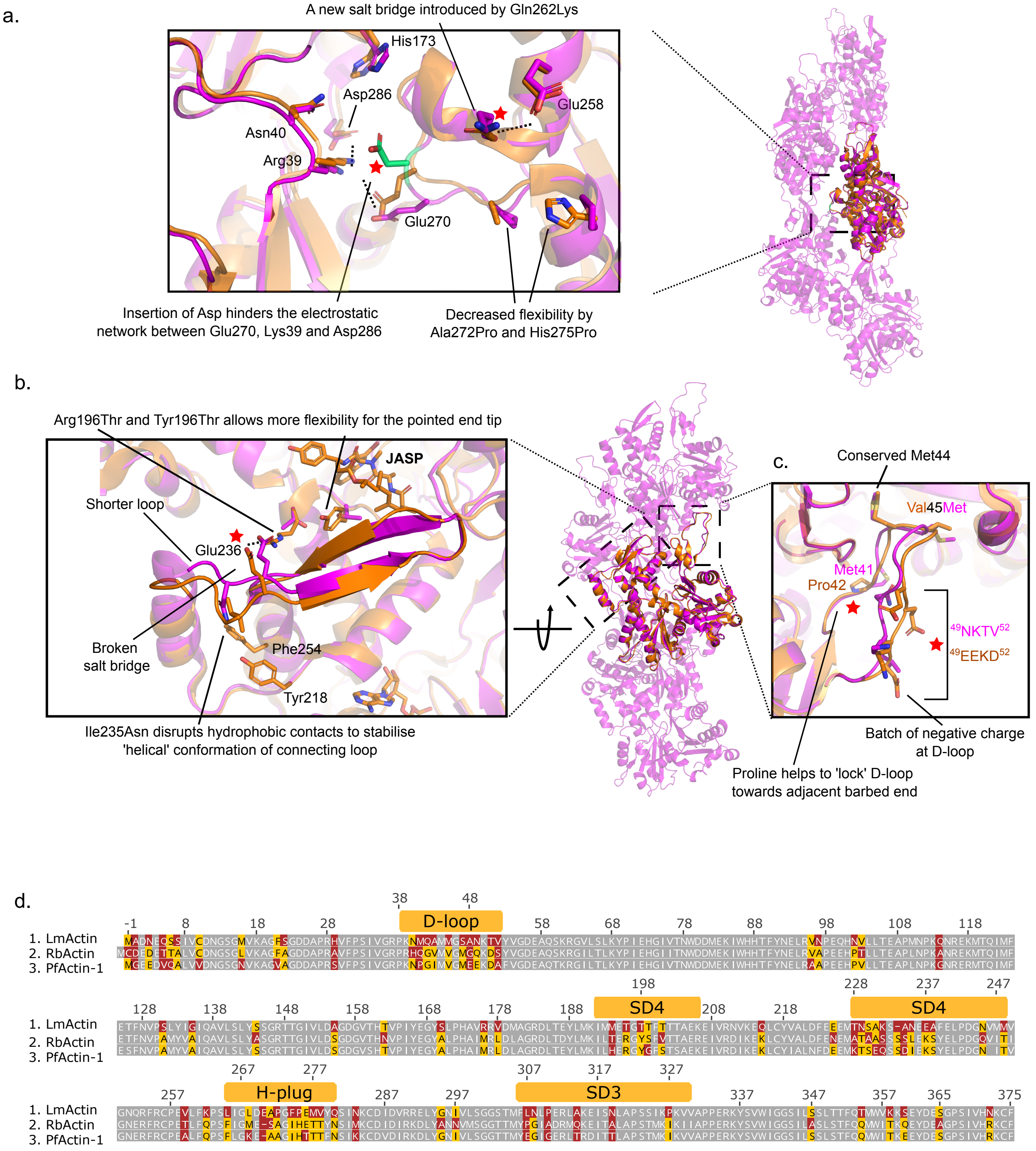
a.
A new salt bridge introduced by Gln262Lys
His173
Asp286
Glu258
Asn40
Arg39
Glu270
Insertion of Asp hinders the electrostatic network between Glu270, Lys39 and Asp286
Decreased flexibility by Ala272Pro and His275Pro
b.
Arg196Thr and Tyr196Thr allows more flexibility for the pointed end tip
JASP
Shorter loop
Glu236
Broken salt bridge
Phe254
Tyr218
Ile235Asn disrupts hydrophobic contacts to stabilise 'helical' conformation of connecting loop
c.
Conserved Met44
Val45Met
Met41
Pro42
49NKTV52
49EEKD52
Batch of negative charge at D-loop
Proline helps to 'lock' D-loop towards adjacent barbed end
d.
D-loop
SD4
SD4
H-plug
SD3
1. LmActin
2. RbActin
3. PfActin-1

Supplementary Fig. 8. Comparison of the actin filament subunit interfaces between *Plasmodium* and *Leishmania* actins. (**a**) H-plug of LmActin (magenta) contains an insertion of an additional aspartate (highlighted in green) in the core of the filament that is not present in malaria actin (orange). Pro272 and Pro275, preceding the H-plug in LmActin, may also alter the flexibility of the H-plug region in comparison with *Plasmodium* actin (PDB 6tu4). Lys263 (absent in *Plasmodium* actin) can form a salt bridge with Glu258 located in the pointed end helix of subdomain 4, and thus link the dynamics of H-plug region to the pointed end of the monomer. Numbering corresponds to LmActin. (**b**) The pointed end of LmActin subdomain 4 contains a single amino acid deletion leading to a shorter loop between α-helix 222-233 and β-strand 238-242. In *Plasmodium* actin, this loop can adopt a different conformation that stabilizes the upstream pointed end tip. In *Plasmodium* actin, Ile236, which is replaced by Asn in LmActin, is inserted into the hydrophobic pocket formed by Tyr218 and Phe255. Furthermore, the Arg196Thr and Tyr196Thr substitutions in LmActin allow more room for the flexibility of the pointed end tip of *Leishmania* actin. Please note that *Plasmodium* actin is stabilized by the presence of jasplokinolide (JASP) drug at this site. Numbering corresponds to LmActin. (**c**) The D-loops of LmActin and *Plasmodium* actin are different. While the Met44 is conserved, malaria actin does not contain a second methionine in position 45. In contrast, Pro42 in *Plasmodium* actin might help to lock the D-loop into an upright position to be inserted into the adjacent barbed end. Interestingly *Plasmodium* actin D-loop has a large batch of negative residues, which might have an impact on the dynamics of the D-loop. (**d**) Sequence comparison between LmActin, rabbit actin, and malaria actin highlighting the singularity of LmActin from the two others.

Supplementary Table 1. Cryo-EM data collection, refinement and validation statistics

	**ADP-Pi actin**	**ADP-actin**	**Cofilin-decorated ADP-actin**
**Microscopy**			
Microscope	Talos Arctica	Titan Krios G2	
Voltage (kV)	200	300	
Target defocus range (µm)	-0.8 to -3.0	-0.75 to -2.5	
Pixel size (Å)	0.97	0.86	
**3D Refinement**			
Number of helical segments	89,543 (239,463)	232,527 (338,734)	46,929 (110,227)
Final resolution (Å)	3.3	2.7	3.4
FSC threshold	0.143	0.143	0.143
Map sharpening ($Å^2$) by Relion	-123.1	-72.3	-92.8
Helical rise (Å)	27.77	28.40	28.56
Helical twist (°)	-166.61	-166.54	-161.26
**Atomic model statistics**			
Chains	5	5	10
Non-hydrogen atoms (waters)	14640	14975 (360)	19685
Proteins residues	1850	1850	2485
Ligands (ADP, Mg2+, PO4)	5, 5, 5	5, 5, 0	5, 5, 0
Molprobity score	1.30	1.09	1.15
Clashscore	3.79	1.69	3.37
Bond RMSD (Å)	0.003	0.005	0.002
Angle RMSD (°)	0.502	0.618	0.442
Poor rotamers (%)	0	0	0.23
Ramachandran favored (%)	97.28	97.01	97.92
Ramachandran allowed (%)	2.72	2.99	2.08
Ramachandran outliers (%)	0	0	0

Supplementary Table 2. Summed PISA energies for actin filament interfaces

Structure (filament in ADP-state)	PDB code	dG (kcal/mol)
Chicken muscle actin	6djo	-22.0
JASP-stabilized PfAct1	6tu4	-21.2
Phalloidin-stabilized rabbit muscle actin	6t20	-24.3
Leishmania actin	7q8c	-17.4

Supplementary Table 3. Plasmid constructs, database references (TriTrypDB) and primers used in this study.

Construct	Description	TriTrypDB
pPL1698-pFastBac1-LmActin-B-thymosin-10xHis	Full length Leishmania major actin with 14 aa flexible linker, human b-thymosin, and the 10xHis-tag	LmjF.04.1230
pPL1415-pSUMOck4-LmCofilin	N-terminal 6xHis-tag-SUMO with full length Leishmania major cofilin, lacking the first methionine	LmjF.29.0510
pPL1718-pCoofy3-LmProfilin	N-terminal 6xHis-tag-GST-tag, 3C cleavage site, full length Leishmania major profilin	LmjF.32.0520
pPL1417-pSUMOck4 -LmTwinfilin	N-terminal 6xHis-tag-SUMO with full length Leishmania major twinfilin, lacking the first methionine	LmjF.34.2290
pPL1818- pSUMOck4-mCofilin-d4c	N-terminal 6xHis-tag-SUMO with full length Leishmania major cofilin, lacking the first methionine and last four C-terminal residues	pPL1415

Primer	Sequence (5'->3')	Description	Construct
pFastBac1_F	TAATAACTCGAGGCATGCGGTACCAA	vector	pPL1698
pFastBac1_R	GAAGCATTTGTTGTGGACGATGGAAGG		
LmCof_F	AGCAGCAGACGGGAGGGGCAATCAGTGG AGTAACATTGGAAGAATC	insert	pPL1415
LmCof_R	CTTTGTTAGCAGCCGGATCTCACTAAACAC TGCGGTGCAAGCG		
SUMO_F	TGAGATCCGGCTGCTAACAAAGCCC	vector	pPL1415, pPL1417
SUMO_R	CCCTCCCGTCTGCTGCTGGA		
Coofy3_v_R	AGGACCCTGGAACAGAACTTCCAGATCC	vector	pPL1718
Coofy_v_F	TGAGATCCGGCTGCTAACAAAGC		
LmProfilin_C18_F	CTGGAAGTTCTGTTCCAGGGTCCTTCCTGG CAGGCATATGTGGATGATTC	insert	
LmProfilin_C18_R	TTTGTTAGCAGCCGGATCTCAGTAGCCAA GTTTGATTAAATAGTCGGCGATAC		
LmTwinfilin_F	AGCAGCAGACGGGAGGGCTTCATATTGAC TTCTGTATTGCCGCTG	insert	pPL1417
LmTwinfilin_R	CTTTGTTAGCAGCCGGATCTCATCAAATCA ACATTGAATGTCCTTTTGGTGC		
LmCof_d4c_F	TTCGCTTGTAGCGCAGTGTTTAGTGAGATC CGGCTG	mutagenesis	pPL1415
LmCof_d4c_R	TAAACACTGCGCTACAAGCGAACCTTACG GATGATTTCCTCG	mutagenesis	pPL1415
