# Supplementary material for: Structural basis of rapid actin dynamics in the evolutionarily divergent Leishmania parasite
Source: Nat Commun. 2022 Jun 15;13:3442. doi: 10.1038/s41467-022-31068-y (PMC9200798; doi:10.1038/s41467-022-31068-y)
Supplement: Supplementary file 3 — Reporting Summary [file 41467_2022_31068_MOESM3_ESM.pdf]

nature portfolio

Corresponding author(s): Pekka Lappalainen
Last updated by author(s): May 20, 2022

Reporting Summary

Nature Portfolio wishes to improve the reproducibility of the work that we publish. This form provides structure for consistency and transparency in reporting. For further information on Nature Portfolio policies, see our Editorial Policies and the Editorial Policy Checklist.

Statistics

For all statistical analyses, confirm that the following items are present in the figure legend, table legend, main text, or Methods section.

n/a	Confirmed	
☐	☒	The exact sample size (*n*) for each experimental group/condition, given as a discrete number and unit of measurement
☐	☒	A statement on whether measurements were taken from distinct samples or whether the same sample was measured repeatedly
☐	☒	The statistical test(s) used AND whether they are one- or two-sided<br>*Only common tests should be described solely by name; describe more complex techniques in the Methods section.*
☒	☐	A description of all covariates tested
☒	☐	A description of any assumptions or corrections, such as tests of normality and adjustment for multiple comparisons
☐	☒	A full description of the statistical parameters including central tendency (e.g. means) or other basic estimates (e.g. regression coefficient) AND variation (e.g. standard deviation) or associated estimates of uncertainty (e.g. confidence intervals)
☐	☒	For null hypothesis testing, the test statistic (e.g. F, t, r) with confidence intervals, effect sizes, degrees of freedom and P value noted<br>*Give P values as exact values whenever suitable.*
☒	☐	For Bayesian analysis, information on the choice of priors and Markov chain Monte Carlo settings
☒	☐	For hierarchical and complex designs, identification of the appropriate level for tests and full reporting of outcomes
☒	☐	Estimates of effect sizes (e.g. Cohen's d, Pearson's r), indicating how they were calculated

Our web collection on statistics for biologists contains articles on many of the points above.

Software and code

Policy information about availability of computer code

Data collection	The cryoEM data was collected using the provided microscope EPU software (Thermo Scientific, version 1.20.3.10).
Data analysis	All steps of the cryoEM image processing were performed in Relion 3.1 software. Movie frames were first aligned with MotionCor2 (v. 1.2.6) or Relion's own implementation of motion correction and CTF estimated by CTFFIND 4.1. Actin filaments were manually picked or 2D class averages from manually picked filaments were used as search templates. FSC and local resolutions were estimated in Relion 3.1. Phenix 1.19.2 'AutoSharpen' was used to produce the final map for model building in Chimera (1.14 build 42094), Coot (0.8.9.1), Namdinator (https://namdinator.au.dk), iSolde (1.1.0) in ChimeraX (1.1.1). Pymol 2.3.0 was used to perform actin filament model analyses with function 'super'. The image analysis for in vitro TIRF experiments was performed manually, with standard functions and packages (in Python: numpy, scipy (curve_fit function), panda, lifelines,).

For manuscripts utilizing custom algorithms or software that are central to the research but not yet described in published literature, software must be made available to editors and reviewers. We strongly encourage code deposition in a community repository (e.g. GitHub). See the Nature Portfolio guidelines for submitting code & software for further information.

Data

Policy information about availability of data

All manuscripts must include a data availability statement. This statement should provide the following information, where applicable:

- Accession codes, unique identifiers, or web links for publicly available datasets
- A description of any restrictions on data availability
- For clinical datasets or third party data, please ensure that the statement adheres to our policy

The cryo-EM reconstructions generated in this study of the L. major ADP-actin filament, ADP-Pi-actin filament and ADP-actin filament - cofilin complex have been

deposited in the Electron Microscopy Data Bank (EMDB) under ID accession codes EMD-13864 [https://www.ebi.ac.uk/emdb/EMD-13864], EMD-13863 [https://www.ebi.ac.uk/emdb/EMD-13863] and EMD-13865 [https://www.ebi.ac.uk/emdb/EMD-13865], respectively. The corresponding atomic models generated in this study have been deposited in the Protein Data Bank (PDB) under accession codes 7Q8C [http://doi.org/10.2210/pdb7Q8C/pdb], 7Q8B [http://doi.org/10.2210/pdb7Q8B/pdb] and 7Q8S [http://doi.org/10.2210/pdb7Q8S/pdb], respectively. The coordinates corresponding to the muscle actin filament structures, cofilin-decorated actin filament structure, yeast cofilin and malaria actin filament structure shown in this article are available from PDB under accession codes 6DJO [http://doi.org/10.2210/pdb6DJO/pdb], 6DJN [http://doi.org/10.2210/pdb6DJN/pdb], 5YU8 [http://doi.org/10.2210/pdb5YU8/pdb], 1QPV [http://doi.org/10.2210/pdb1qpv/pdb] and 6TU4 [http://doi.org/10.2210/pdb6TU4/pdb], respectively. The sequence data used in this study was obtained from Uniprot and the accession codes are described in Source Data file. Source data for the biochemical experiments using TIRFM data are provided in Source Data File. Other data are available from the corresponding author upon reasonable request.

Field-specific reporting

Please select the one below that is the best fit for your research. If you are not sure, read the appropriate sections before making your selection.

[x] Life sciences [ ] Behavioural & social sciences [ ] Ecological, evolutionary & environmental sciences

For a reference copy of the document with all sections, see nature.com/documents/nr-reporting-summary-flat.pdf

Life sciences study design

All studies must disclose on these points even when the disclosure is negative.

Sample size	Actual sample size calculation does not apply to any experiments carried out in this study. However, for all biochemical assays sufficient amount of data were collected for statistical analysis. More specifically, when the measure over a single experiment is obtained as an average, for example the polymerization rate, we analyzed approximately 20 filaments per experiment. All filaments were exposed to same solution and thus polymerize at the same rate. When the measure over a single experiment was obtained from a fit, for example severing, the fit was performed over approximately 50 filaments. The reason is that only one event occurs per filaments - unlike polymerization where hundreds of binding events occur per filament. When comparing two conditions, e.g. LmActin vs RbActin, the experiment was repeated at least 3 times, on at least 2 different days. When looking at the impact of a continuous variable, e.g. cofilin and Twf concentration, the experiment was repeated at least 5 times over a range of concentrations.
Data exclusions	In the TIRF microscopy experiments determining actin filament dynamics, the filaments that would stick to the surface or exhibited photo-induced pauses were excluded from the analysis of different experiments (ref: Niedermayer et al 2012, PNAS). In cryoEM data analysis, micrographs that were poorly aligned, or with poor CTF assessment, or contained major dirt or contaminants were excluded. Filaments were manually picked from central parts of the filament (excluding broken, bent, overlapped or ends of the filaments) to train automated picking for ADP-Pi sample. For ADP-actin and cofilin-decorated sample, actin filaments were manually picked as described for ADP-Pi sample. In the preceding 2D and 3D classification steps, only the 'particles' producing sharp classes were included in further analysis.
Replication	We show all data from different experiments in the data plots presented in the figures. The figures are coloured to describe the data from different experiments.
Randomization	In TIRF experiments, the filaments were picked randomly on the first frames to avoid bias. For structural biology (cryoEM), randomization is not relevant due to the type of obtained data.
Blinding	Investigators were not blinded for any of the analyses. This was not possible since data collection and analysis was performed by the same investigator.

Reporting for specific materials, systems and methods

We require information from authors about some types of materials, experimental systems and methods used in many studies. Here, indicate whether each material, system or method listed is relevant to your study. If you are not sure if a list item applies to your research, read the appropriate section before selecting a response.

Materials & experimental systems

n/a	Involved in the study	
[x]	[ ]	Antibodies
[ ]	[x]	Eukaryotic cell lines
[x]	[ ]	Palaeontology and archaeology
[x]	[ ]	Animals and other organisms
[x]	[ ]	Human research participants
[x]	[ ]	Clinical data
[x]	[ ]	Dual use research of concern

Methods

n/a	Involved in the study	
[x]	[ ]	ChIP-seq
[x]	[ ]	Flow cytometry
[x]	[ ]	MRI-based neuroimaging

Eukaryotic cell lines

Policy information about cell lines

Cell line source(s)	ExpiSF9 was purchased from Thermo Scientific.
Authentication	The cell line was commercially purchased and thus not authenticated in our laboratory.
Mycoplasma contamination	This is a commercial cell-line from Thermo Scientific, where possible mycoplasma contamination was tested. Moreover, the cell-line was used only as a tool for protein production.
Commonly misidentified lines (See ICLAC register)	*Name any commonly misidentified cell lines used in the study and provide a rationale for their use.*
